# Characterization of New Polyol/H^+^ Symporters in *Debaryomyces hansenii*

**DOI:** 10.1371/journal.pone.0088180

**Published:** 2014-02-04

**Authors:** Iliana Pereira, Ana Madeira, Catarina Prista, Maria C. Loureiro-Dias, Maria José Leandro

**Affiliations:** 1 Centre for Botany Applied to Agriculture (CBAA), Instituto Superior de Agronomia, University of Lisbon, Lisbon, Portugal; 2 Research Institute for Medicines and Pharmaceutical Sciences (iMed.UL) and Department of Biochemistry and Human Biology, Faculty of Pharmacy, University of Lisbon, Lisbon, Portugal; University of Florida, United States of America

## Abstract

*Debaryomyces hansenii* is a halotolerant yeast that produces and assimilates a wide variety of polyols. In this work we evaluate polyol transport in *D. hansenii* CBS 767, detecting the occurrence of polyol/H^+^ (and sugar/H^+^) symporter activity, through the transient extracellular alkalinization of unbuffered starved cell suspensions. From the *D. hansenii* genome database, we selected nine ORFs encoding putative transporter proteins to clone in a centromeric plasmid with C-terminal GFP tagging and screened for polyol/H^+^ symporters by heterologous expression in *Saccharomyces cerevisiae*. Five distinct *D. hansenii* polyol/H^+^ symporters were identified and characterized, with different specificities and affinities for polyols, namely one glycerol-specific (*Dh*Stl1), one D-galactitol-specific (*Dh*Sgl1, Symporter galactitol/H^+^ 1), one D-(+)-*chiro*-inositol-specific (*Dh*Syi1, Symporter D-(+)-*chiro*-inositol/H^+^ 1), one for D-sorbitol/D-mannitol/ribitol/D-arabitol/D-galactitol (*Dh*Syl1, Symporter Polyols 1) and another for D-sorbitol/D-mannitol/ribitol/D-arabitol (*Dh*Syl2, Symporter Polyols 2). This work contributed to the annotation of new yeast polyol transporters, including two specific for uncommon substrates as galactitol and D-(+)-*chiro*-inositol.

## Introduction

In response to salt- and drought-stress, fungi, marine algae and vascular plants can synthesize and accumulate soluble compounds with low molecular weight. These compounds are designated as compatible solutes because they are compatible with cell metabolism, even when they accumulate at high intracellular concentrations. Compatible solutes include sugar alcohols, quaternary ammonia compounds, proline and tertiary sulfonic compounds. Sugar alcohols or polyols (such as glycerol, mannitol and sorbitol) correspond to the chemically reduced form of an aldose or a ketose. Being more reduced, polyols are higher energy storage molecules than their corresponding sugars (*e.g.* mannitol *versus* mannose). It has been suggested that polyols may mimic the structure of water and maintain an artificial sphere of hydration around macromolecules. They may also act as scavengers of reactive oxygen species, thereby preventing peroxidation of lipids that would cause cell damage [1].

Polyols have several applications in the food, pharmaceutical, medical and chemical industries, as food additives, texturizing agents, low-caloric sweeteners, pharmaceutical formulating agents, in the manufacture of intravenous fluids and precursors of many synthetic polymers and resins [2]–[7].

Industrial production of most polyols is performed by catalytic reduction of sugars with hydrogen gas and nickel at high temperature and pressure, what requires highly pure sugar substrates and costly chromatographic purification steps. Lately, processes using bacteria (specially lactic acid bacteria) and yeasts have demonstrated that biotechnological production may represent an efficient and cost-effective alternative to the chemical production of polyols [8].

The polyols most frequently found in plants are the derivatives of glucose (sorbitol) and mannose (mannitol). The first plant polyol transporter characterized at the molecular level was the mannitol/H^+^ symporter *Ag*MaT1 from celery (that also seems to transport xylitol and sorbitol) [9]. Since then, several other polyol transporters have been characterized in plants, such as the broad-spectrum H^+^-symporter *At*Plt5 (*At*Pmt5) from *Arabidopsis thaliana* (that besides linear polyols, as sorbitol, xylitol, erythritol and glycerol, also transports *myo*-inositol and different hexoses and pentoses, including ribose) [10], the *Hb*Plt2 xylitol/H^+^ symporter (that also seems to transport the cyclic polyol quebrachitol) from rubber tree [11] and the specific xylitol/H^+^ symporter *Lj*Plt4 from the model legume *Lotus japonicus* [12]. These polyol transporters are members of the Sugar Porter Family of the Major Facilitator Superfamily (MFS) [13], although they are not closely related to known sucrose and hexose transporters [14]. As most MFS proteins, polyol transporters are integral membrane proteins with 12 membrane-spanning domains and the consensus sequences of the Sugar Porter Family [15] are also generally conserved in polyol transporters [12], [14].

Biochemical characterization of polyol transport and metabolism in yeasts has been poorly studied so far. However, the ability of yeasts to use (or not to use) polyols as carbon source has been always used by taxonomists to characterize yeast species, showing that polyols are indeed important yeast substrates. Using taxonomical data [16], we analized the ability to assimilate polyols of 464 ascomycete and 216 basidiomycete yeast species. The main outcome of this analysis is that most yeasts assimilate at least one polyol. Ascomycete yeasts assimilate preferably glycerol, then sorbitol and mannitol, whereas in basidiomycete yeasts the order of preference is mannitol, sorbitol and glycerol (Table 1). It is remarkable that more than 10% of all the species under analysis can assimilate the four polyols arabitol, ribitol, sorbitol and xylitol.

**Table 1:** Prevalence of polyol assimilation by yeasts (based on [16]).

Polyols	% Ascomycetes	% Basidiomycetes
Glycerol	73.6	54.8
D-Sorbitol	66.1	77.4
D-Mannitol	65.6	85.2
D-Xylitol	39.3	33.6
Ribitol	37.6	46.5
meso-Erythritol	23.3	27.6
L-Arabitol	19.4	21.6
D-Galactitol	6.3	21.2
*myo*-Inositol	6.0	38.7

The existence of polyol transporters was detected in several yeasts. In *Rhodotorula glutinis* a constitutive active transport system for pentitols and erythritol, and another carrier for ribitol and L-arabitol were reported [17], [18]. In *Candida intermedia* it was shown that sorbitol was transported by a high affinity (K_m_ = 6 mM) polyol/H^+^ symporter, which accepted also xylitol, D-arabitol and D-mannitol, but not glucose. In this yeast, sorbitol was also transported by a glucose/H^+^ symporter. In this case the affinity for sorbitol was lower (K_m_ = 200 mM) and the transport was inhibited by glucose and other hexoses [19]. *myo*-Inositol transport was also characterized in *C. albicans* as a proton transporter system [20].

So far, only glycerol and *myo*-inositol transporters have been characterized at the molecular level in yeasts. The Stl1 glycerol/H^+^ symporter was first characterized in *Saccharomyces cerevisiae* [21]. This transporter was also described in *C. albicans* [22] and detected in several yeasts as *Debaryomyces hansenii* and *Zygosaccharomyces rouxii* [23], [24]. Two *myo*-inositol transporters were described in *S. cerevisiae*, Itr1 (the major permease for *myo*-inositol) and Itr2 [25]. In *Schizosaccharomyces pombe* the genes involved in inositol transport (*ITR1* and *ITR2*) are required for mating and sporulation [26].

*D. hansenii* is a halotolerant yeast usually found in salty environments, such as the sea and salted food. This yeast is capable of accumulating sodium without being intoxicated even when potassium is present at low concentration in the environment, and sodium improves *D. hansenii* growth [27] and protects this yeast in the presence of additional stress factors such as high temperature and extreme pH [28]. *D. hansenii* is also a polyol producing yeast that can have a potential use for increasing value of lignocellulosic hydrolysates by xylitol [29] and arabitol production [30].

The availability of the genome of *D. hansenii* CBS 767 by Génolevures consortium [31] opened the possibility of molecular studies of this yeast and the identification of proteins with interesting properties. In the phylogenetic analysis of protein members of the Sugar Porter family from eight sequenced yeasts, Palma *et al* reported the existence of twenty two *D. hansenii* proteins with undetermined substrate, besides eight putative glycerol transporters [32].

Taking into account that the yeasts from the genus *Debaryomyces* are among the ascomycete yeasts that assimilate a broader array of polyols [16] and that *D. hansenii* CBS 767 complete genome sequence is available, we studied polyol transport in more detail in this yeast. In this work, we analyzed polyol/H^+^ transport in *D. hansenii*, screening for putative polyol/H^+^ symporters by heterologous expression in *S. cerevisiae* of nine putative transporter proteins, five of which we characterized at the biochemical level as new *D. hansenii* polyol/H^+^ symporters.

## Materials and Methods

### Strains and growth media

The yeast strains used or generated in this work are listed in Table 2. Yeast strains were grown on minimal YNB (yeast nitrogen base without amino acids containing the indicated carbon source and the required supplements) or in rich YPD (1% yeast extract, 2% peptone, 2% glucose) media. Cultures were incubated at 28°C with shaking. Sugar and polyol concentrations are given as percentages (w/v). *Escherichia coli* strain DH5α (Stratagene) was used as the host for plasmid amplification. *E. coli* transformants were grown on standard Luria–Bertani medium supplemented with ampicillin (100 µg ml^−1^).

**Table 2:** Strains used or generated in this work.

Strain	Description	Reference
*D. hansenii* CBS 767 (var. *hansenii*)	Type strain	CBS strain database
*S. cerevisiae* YSH 1172	10560-6B *aqy1*::*kanMX4 aqy2*::*HIS3 MATα leu2*::*hisG trp1*::*hisG his3*::*hisG ura3-52 aqy1Δ*::*kanMX4 aqy2*::*HIS3*	[53]
**MLY10**	YSH 1172+pUG35	This work
**MLY7**	YSH 1172+pUG35-DEHA2C06380*g*	This work
**MLY8**	YSH 1172+pUG35-DEHA2E01386*g*	This work
**MLY9**	YSH 1172+pUG35-DEHA2C05896*g*	This work
**MLY12**	YSH 1172+pUG35-DEHA2C05918*g*	This work
**MLY20**	YSH 1172+pUG35-DEHA2F15444*g*	This work
**MLY22**	YSH 1172+pUG35-DEHA2E24310*g*	This work
**MLY23**	YSH 1172+pUG35-DEHA2G06490*g*	This work
**MLY25**	YSH 1172+pUG35-DEHA2E00726*g*	This work
**MLY26**	YSH 1172+pUG35-DEHA2B00528*g*	This work

### Plasmid and strain construction

*D. hansenii* genomic DNA for PCR amplification was isolated as described by Cryer *et al.* [33] after a previous treatment with lyticase (5 mg ml^−1^).

The plasmid used for cloning was pUG35 (AF298787, NCBI nucleotide database). The complete DEHA2C06380*g* ORF was amplified with high-fidelity DNA polymerase Phusion F-530 (Finnzymes) using primers Dh6380FOR and Dh6380REV (Table 3). Both primers contain, respectively, *Xba*I and *Sal*I restriction sites at the 5′ end. Plasmid pUG35 was digested with *Xba*I and *Sal*I and ligated to the DEHA2C06380*g* ORF fragment, previously digested with the same restriction enzymes, resulting in plasmid pUG35-DEHA2C06380*g*. A similar procedure was also used to clone the DEHA2E01386*g*, DEHA2C05896*g*, DEHA2C05918*g* and DEHA2F15444*g* ORFs into plasmid pUG35 (see Table 3), behind *MET25* promoter and in frame with GFP sequence and CYC1-T terminator. All constructed plasmids and control plasmid pUG35 were transformed into *S. cerevisiae* YSH 1172 using the LiAc method [34]. DEHA2E24310*g* ORF (amplified by PCR with primers Dh24310FOR and Dh24310REV) was inserted behind the *MET25* promoter of pUG35 plasmid (previously digested with *Xba*I and *Sal*I) by homologous recombination [35] directly into *S. cerevisiae* YSH 1172, resulting in strain MLY22. The same procedure was also used to clone the DEHA2G06490*g*, DEHA2E00726*g* and DEHA2B00528*g* ORFs (see Table 3).

**Table 3:** Primers used in this work (restriction sites in primers used to clone by restriction digestion and ligation are in bold; sequences homologous to the *D. hansenii* genes cloned by homologous recombination are underlined).

Gene to amplify	Primer name	Sequence (5′- 3′)	Restriction enzymes used for cloning	Constructed *S. cerevisiae* strain
**DEHA2C06380** ***g***	Dh6380FOR	CTCAGC**TCTAGA**CGATTGAACATGAGTAATTCTAC	*Xba*I and *Sal*I	**MLY7**
	Dh6380REV	ATCTAT**GTCGAC**TGCACTGTGATCGTCATTTTGAG		
**DEHA2E01386** ***g***	Dh1386FOR	CACCTG**TCTAGA**AATAATATGTATAAAATATGGTC	*Xba*I and *Sal*I	**MLY8**
	Dh1386REV	AATACT**GTCGAC**AACTTCCGCAGGCTTAACTG		
**DEHA2C05896** ***g***	Dh5896FOR	CTGGAA**TCTAGA**GGAACTAAAATGTCTTCAAGTGC	*Xba*I and *Sal*I	**MLY9**
	Dh5896REV	ACTACT**GTCGAC**CTCAATATGAGCAGTTTCG		
**DEHA2C05918** ***g***	Dh5918FOR	AGCAGT**TCTAGA**ATTATGCCTTCAGAAACTAATCC	*Xba*I and *Sal*I	**MLY12**
	Dh5918REV	CACTTA**GTCGAC**CTCGATATGAGCAGTTTCG		
**DEHA2F15444** ***g***	Dh15444FOR	GCACCA**ACTAGT**TTCAACTCAATAATCAAAATGTATG	*Spe*I and *Sal*I	**MLY20**
	Dh15444REV	TATTAT**GTCGAC**GTTCCTTTTAAAGAATCGCAAAGC		
**DEHA2E24310** ***g***	Dh24310FOR	TAGATACAATTCTATTACCCCCATCCATACTCTAGAACTAGTATGAAAAACAACATAGATGGTACG		**MLY22**
	Dh24310REV	ACCAGTGAATAATTCTTCACCTTTAGACATGTCGAGGTCGACCGATATACCTTCTTCGTGTAAAATTGTGG		
**DEHA2G06490** ***g***	Dh6490FOR	TAGATACAATTCTATTACCCCCATCCATACTCTAGAACTAGTATGGCAACAGAAGACAAAGAACTCGGAGTCG		**MLY23**
	Dh6490REV	ACCAGTGAATAATTCTTCACCTTTAGACATGTCGAGGTCGACAACAGTTTCTATATGCCCAACTTCAGG		
**DEHA2E00726** ***g***	Dh0726FOR	TAGATACAATTCTATTACCCCCATCCATACTCTAGAACTAGTATGAAGATAGTGATAATAAGGGACATTATGG		**MLY25**
	Dh0726REV	ACCAGTGAATAATTCTTCACCTTTAGACATGTCGAGGTCGACGTTTTTATCTACTTTCTCTTCAATGTGTTCAAC		
**DEHA2B00528** ***g***	Dh0528FOR	TAGATACAATTCTATTACCCCCATCCATACTCTAGAACTAGTATGACCGAGAAAGTTGATATGATTGGTACG		**MLY26**
	Dh0528REV	ACCAGTGAATAATTCTTCACCTTTAGACATGTCGAGGTCGACAATTTCCTCGACATGAAGAGTTTCATTAG		

### Microscopy

For the visualization of GFP-tagged transporters, mid-exponential phase cells were observed with a Leitz Wetzlar Germany 513558 epifluorescence microscope equipped with a Leitz Wetzlar Germany Type 307-148002 514687 mercury bulb and a BP 340-380; BP 450–490 (for GFP visualizing); BP 515–560 filter set. Images were obtained with a digital camera Axiocam Zeiss.

### Symport assays

The detection of H^+^ movements associated with initial sugar/polyol uptake was assessed by adding sugar/polyol pulses to unbuffered cell suspensions, as described in [36], [37]. If the added sugar/polyol is transported to the interior of the cells by a mechanism of symport with H^+^ an extracellular alkalinization occurs. If there is no alkalinization it means that the added sugar/polyol is not transported by a H^+^ symport mechanism. *D. hansenii* cultures (OD_640_ of 0.9–1.2) and *S. cerevisiae* cultures (OD_640_ of 0.7–0.9) were harvested by centrifugation (10 000 *g*, 5 min, 4°C), washed twice with ice-cold water, resuspended in water to a final concentration of about 25 mg (dw) ml^−1^ and kept on ice for at least one hour. Kinetic parameters of polyol transporters were determined testing polyol concentrations in the range 0.5 to 400 mM, calculating the slope of initial extracellular alkalinization for each assay and kinetic parameters were estimated using GraphPad Prism version 5.00 (Graphpad Software, www.graphpad.com) for Michaelis-Menten regression analysis. A stoichiometry of one H^+^ per polyol was assumed.

In assays performed after starvation, the cells were washed once with cold sterile water and incubated in the same volume of YNB medium without carbon source for 3 hours or overnight. When indicated, KCl to a final concentration of 1 M was added to the aqueous cell suspension.

For *S. cerevisiae* constructed strains, the detection of the maltose/H^+^ symporter was used as a positive control (cells grown on maltose). Twelve polyols (2,3-butanediol, D-arabitol, L-arabitol, i-erythritol, D-galactitol, glycerol, D-(+)-*chiro*-inositol, *myo*-inositol, D-mannitol, ribitol, D-sorbitol, D-xylitol) and seven sugars (L-arabinose, D-fructose, D-galactose, D-glucose, D-ribose, L-sorbose, D-xylose) were tested for extracellular alkalinization.

### Sugar transport assays

Initial [U-^14^C] sorbitol (GE Healthcare formerly Amersham Biosciences) uptake rates and inhibition assays were performed as described in [38]. Cultures were harvested at OD_640_∼0.8. To test inhibition, 1.5 mM [U-^14^C] sorbitol uptake was performed in the presence of 20 mM mannitol.

Accumulation ratios were calculated for [U-^14^C] sorbitol as described in [19]. The measurements were performed at 25°C in small test-tubes, with an initial concentration of 0.1 mM [U-^14^C] sorbitol. When indicated carbonyl cyanide m-chlorophenylhydrazone (CCCP) to a final concentration of 0.25 mM was added.

### Miscellaneous

DNA manipulations were performed according to standard protocols [39]. Restriction enzymes and the ligase were purchased from Roche. Primers were obtained from STAB VIDA (Caparica, Portugal). Plasmid DNA from *E. coli* was isolated using a GenElute™ Plasmid Miniprep Kit (Sigma-Aldrich). Sequencing was performed at STAB VIDA (Caparica, Portugal).

## Results

### Detection of polyol/H^+^ symport activity in *D. hansenii*

**Figure 1:**
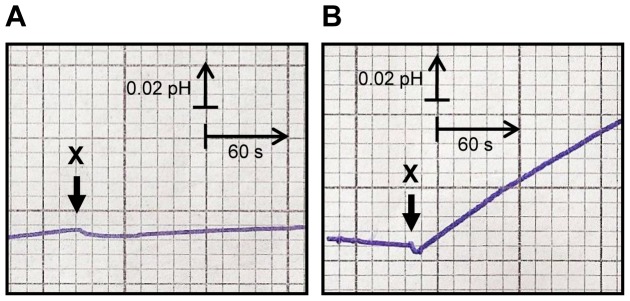
Symport activity in *D. hansenii* CBS 767 with and without starvation. Effect on extracellular pH elicited by addition of 10-xylitol to unbuffered cell suspensions (pH 5, 1 M KCl) of *D. hansenii* CBS 767, grown on YNB medium with 2% xylitol, without starvation (A) and after 3 h starvation (B). The arrows indicate the times of xylitol addition. Data are representative of at least two independent experiments.

Polyol and sugar transport in cells grown on different carbon sources was assessed in *D. hansenii* strain CBS 767. The presence of polyol (or sugar) H^+^ symport activity (detected by external alkalinization of an unbuffered cell suspension after the addition of the polyol/sugar to be tested) was only detected after submitting the cells to 3 h starvation (Figure 1). Addition of 1 M KCl to the cells, before pH adjustment to 5.0, made pH recording data more stable and, consequently, symport signals more evident.

Sugar/H^+^ symport activity for glucose, xylose, galactose and fructose was detected, after 3 h carbon source starvation, when cells were grown on glucose but not when grown on xylitol, glycerol or mannitol. When cells grown on xylitol were submitted to overnight starvation, H^+^ symport activity for those four sugars became detectable (results not shown).

**Figure 2:**
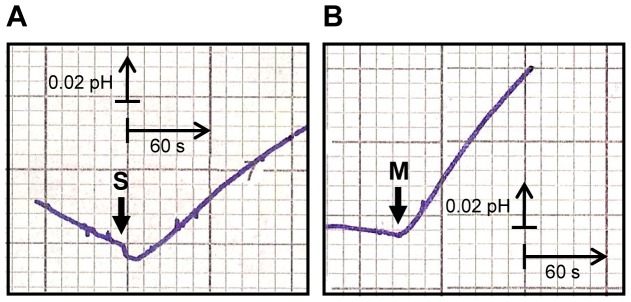
Symport activity in *D. hansenii* CBS 767 after 3 h starvation. Effect on extracellular pH elicited by addition of 10-sorbitol (S, panel A) or D-mannitol (M, panel B) to unbuffered cell suspensions (pH 5, 1 M KCl) of *D. hansenii* CBS 767, grown on YNB with 2% xylitol, after 3 h starvation. The arrows indicate the times of polyol addition. Data are representative of at least two independent experiments.

*D. hansenii* cells displayed polyol/H^+^ symport activity (for xylitol, sorbitol and mannitol), after 3 h carbon starvation, when grown on xylose, glucose, xylitol, glycerol and mannitol media (Figures 1B and 2).

*D. hansenii* cells grown on glucose, after 3 h carbon starvation, also displayed polyol/H^+^ symport signals for D-(+)-*chiro*-inositol, ribitol, D-arabitol and galactitol, but not *myo*-inositol or L-arabitol (results not shown).

### Screening and cloning of putative polyol transporters from *D. hansenii*

To identify *D. hansenii* putative polyol/H^+^ transporters, protein sequences from already identified polyol transporters were obtained from available databases and used as query in a BLASTP search on *D. hansenii* CBS 767 genome (available at Génolevures Consortium Website, http://cbi.labri.fr/Genolevures/).

Nine *D. hansenii* genes encoding proteins displaying the higher sequence homology to characterized polyol transporters were selected (previous name and Gene IDs are indicated): DEHA2C06380*g* (DEHA0C07161*g*; Gene ID: 2900852); DEHA2E01386*g* (DEHA0E01936*g*; Gene ID: 2902914); DEHA2C05896*g* (DEHA0C06655*g*; Gene ID: 8998320); DEHA2C05918*g* (DEHA0C06677*g*; Gene ID: 2900492); DEHA2F15444*g* (DEHA0F16720*g*; Gene ID: 2904159); DEHA2E24310*g* (DEHA0E25839*g*; Gene ID: 2903016); DEHA2G06490*g* (DEHA0G07139*g*; Gene ID: 2904708); DEHA2E00726*g* (DEHA0E00957*g*; Gene ID: 2901972); DEHA2B00528*g* (DEHA0B00517*g*; Gene ID: 2913088).

Specific primers were designed to amplify the corresponding opening reading frames (ORFs) from *D. hansenii* genomic DNA and the amplified ORFs (without the STOP codons) were expressed in the centromeric plasmid pUG35 under the control of the methionine-repressible *MET*25 promoter, with C-terminal GFP tagging.

**Figure 3:**
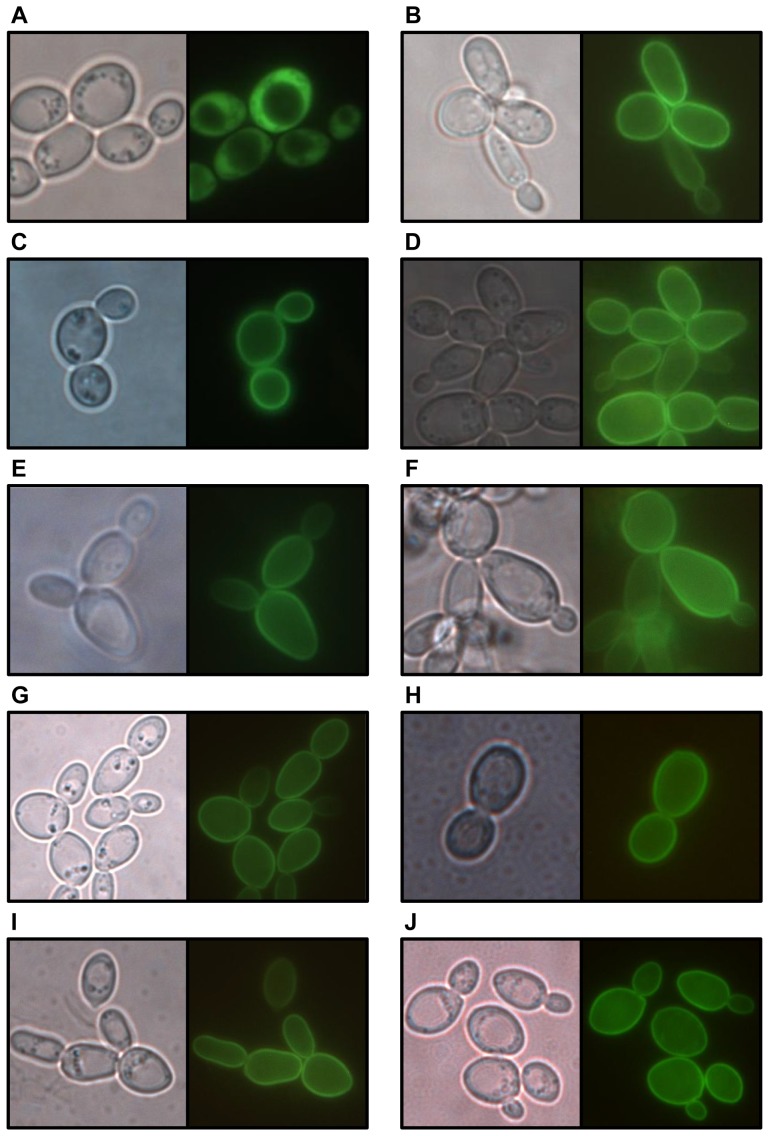
Localization of GFP-tagged *D. hansenii* putative transporters in *S. cerevisiae* plasma membrane. Phase contrast (left panels) and epifluorescence (right panels) images of *S. cerevisiae* YSH 1172 transformed with constructed centromeric plasmids (driven by the *MET25* promoter). Strains MLY10 (A, empty plasmid), MLY7 (B), MLY8 (C), MLY9 (D), MLY12 (E), MLY20 (F), MLY22 (G), MLY23 (H), MLY25 (I), MLY26 (J), grown on YNB medium with 2% maltose.

Constructed plasmids were expressed in *S. cerevisiae* YSH 1172 that was previously shown to be unable to transport sorbitol [40]. Empty plasmid pUG35 was also cloned as a negative control (strain MLY10; Figure 3A). All the nine cloned genes encode proteins located at the plasma membrane, based on GFP fluorescence observation (Figure 3B–J), as would be expected for membrane transporters.

### Characterization of *S. cerevisiae* strains harboring putative polyol transporters from *D. hansenii*

For the *S. cerevisiae* strains harboring *D. hansenii* putative polyol transporters, symport assays were performed. A list of seven sugars and twelve polyols were tested. The presence of maltose/H^+^ symport activity for the *S. cerevisiae* host strain when grown on maltose medium was used as a positive control for H^+^ symport activity in all assays. The results obtained are summarized in Table 4.

**Table 4:** Symport activity detected in the *S. cerevisiae* strains constructed in this work.

S. cerevisiae strain	Description	Symport Activity	K_m_ (mM)	V_max_(mmol h^−1^ (gdw)^−1^)
**YSH 1172**	host strain	-		
**MLY10**	YSH 1172+pUG35	-		
**MLY7**	YSH 1172+pUG35- DEHA2C06380g	-		
**MLY8**	YSH 1172+pUG35-DEHA2E01386g	Glycerol	nd	nd
**MLY9**	YSH 1172+pUG35-DEHA2C05896g	D-Sorbitol	11.51±2.50	2.87±0.32
		D-Mannitol	0.30±0.09	0.47±0.02
		Ribitol	11.44±4.46	0.88±0.11
		D-Arabitol	0.96±0.32	0.44±0.03
		D-Galactitol	12.33±1.50	0.47±0.02
**MLY12**	YSH 1172+pUG35- DEHA2C05918g	D-Sorbitol	13.82±2.12	0.60±0.03
		D-Mannitol	0.93±0.16	0.88±0.03
		Ribitol	18.31±3.23	0.94±0.07
		D-Arabitol	0.46±0.21	0.92±0.06
**MLY20**	YSH 1172+pUG35- DEHA2F15444g	-		
**MLY22**	YSH 1172+pUG35- DEHA2E24310g	D-Galactitol	1.93±0.77	0.22±0.02
**MLY23**	YSH 1172+pUG35- DEHA2G06490g	D-(+)-chiro-Inositol	18.64±9.23	0.05±0.01
**MLY25**	YSH 1172+pUG35- DEHA2E00726g	-		
**MLY26**	YSH 1172+pUG35- DEHA2B00528g	-		

The kinetic transport parameters (K_m_ and V_max_) for the different substrates are shown. No activity was detected for sugars (L-arabinose, D-fructose, D-galactose, D-glucose, D-ribose, L-sorbose, D-xylose).

nd – not determined.

**Figure 4:**
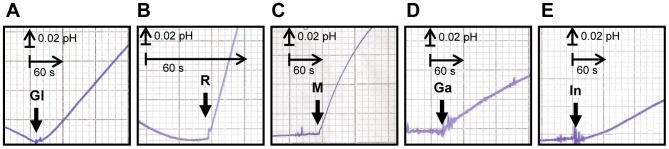
Symport activity in *S. cerevisiae* strains expressing *D. hansenii* polyol transporters. Effect on extracellular pH elicited by addition of 10(Gl), ribitol (R), D-mannitol (M), D-Galactitol (Ga) or D-(+)-*chiro*-inositol (In) to unbuffered cell suspensions (pH 5) of *S. cerevisiae* strains MLY8 (A), MLY9 (B), MLY12 (C), MLY22 (D) and MLY23 (E), grown on YNB medium with 2% maltose. The arrows indicate the times of polyol addition. Data are representative of at least two independent experiments.

Strain MLY8 (YSH 1172+pUG35-DEHA2E01386*g*) displayed only glycerol symport signal (Figure 4A), indicating that the gene DEHA2E01386*g* encodes a *D. hansenii* glycerol/H^+^ symporter. It presents 68% protein homology to *C. albicans* and 64% to *S. cerevisiae* Stl1 glycerol symporters, so we named the corresponding protein as *Dh*Stl1.

Strain MLY9 (YSH 1172+pUG35-DEHA2C05896*g*) displayed D-sorbitol, D-mannitol, ribitol (Figure 4B), D-arabitol and D-galactitol symport signals, indicating that DEHA2C05896*g* encodes a *D. hansenii* sorbitol/mannitol/ribitol/arabitol/galactitol/H^+^ symporter, that was named *Dh*Syl1 (Symporter Polyols 1). In this strain, the addition of mannitol reduced [U-^14^C] sorbitol uptake by 70% (results not shown).

Strain MLY12 (YSH 1172+pUG35-DEHA2C05918*g*) also displayed D-sorbitol, D-mannitol (Figure 4C), ribitol and D-arabitol symport signals, indicating that DEHA2C05918*g* encodes another *D. hansenii* sorbitol/mannitol/ribitol/arabitol/H^+^ symporter, that was named *Dh*Syl2 (Symporter Polyols 2).

**Figure 5:**
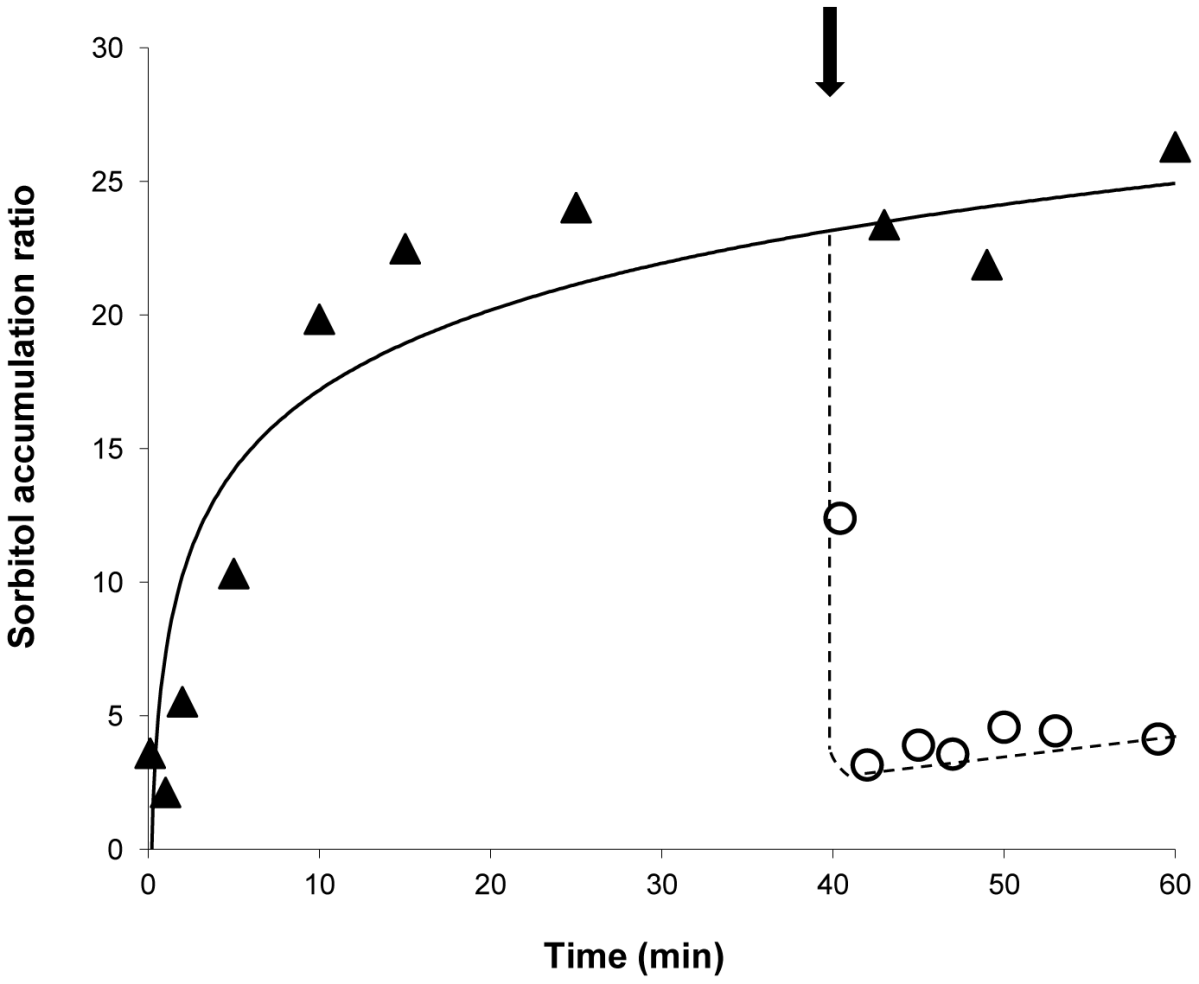
Effect of CCCP on sorbitol accumulation in *S. cerevisiae* MLY9. Accumulation of [U-^14^C] sorbitol (0.1 mM) by *S. cerevisiae* MLY9 cells (▴) grown on maltose medium. The arrow indicates the addition of 0.25 mM CCCP (○).

Sorbitol accumulation in strains MLY9 (Figure 5) and MLY12 (results not shown) was sensitive to the protonophore CCCP, as expected for a H^+^ dependent symport transport mechanism.

Strain MLY22 (YSH 1172+pUG35-DEHA2E24310*g*) displayed D-galactitol symport signals (Figure 4D), indicating that DEHA2E24310*g* encodes a *D. hansenii* CBS 767 galactitol/H^+^ symporter, that was named *Dh*Sgl1 (Symporter galactitol/H^+^).

Strain MLY23 (YSH 1172+pUG35-DEHA2G06490*g*) displayed D-(+)-*chiro*-inositol symport signals (Figure 4E), indicating that DEHA2G06490*g* encodes a *D. hansenii* CBS 767 D-(+)-*chiro*-inositol/H^+^ symporter, that was named *Dh*Syi1 (Symporter D-(+)-*chiro*-inositol/H^+^).

In strains MLY7, MLY20, MLY25 and MLY26 the addition of any of the tested seven sugars or twelve polyols did not trigger initial external alkalinization, indicating that a mechanism of symport with protons for those substrates was not active in these strains.

Alignment of the five characterized polyol transporters (Figure S1) showed that the consensus sequences of the Sugar Porter Family [15] are present and that the glycerol symporter *Dh*Stl1 (549 amino acids) and the D-(+)-*chiro*-inositol symporter *Dh*Syi1 (536 amino acids) are more dissimilar to the other three symporters, being almost 100 amino acids shorter. The galactitol/H^+^ symporter *Dh*Sgl1 (626 amino acids) has 65% homology to *Dh*Syl1 (649 amino acids) and to *Dh*Syl2 (649 amino acids). *Dh*Syl1 and *Dh*Syl2 have 90% protein homology to each other.

*Dh*Stl1, *Dh*Syi1, *Dh*Syl1 and *Dh*Syl2 probably contain 12 transmembrane hydrophobic domains, whereas *Dh*Sgl1 probably contains only 10 [as predicted by the HMMTOP Server v. 2.0 [41]].

**Figure 6:**
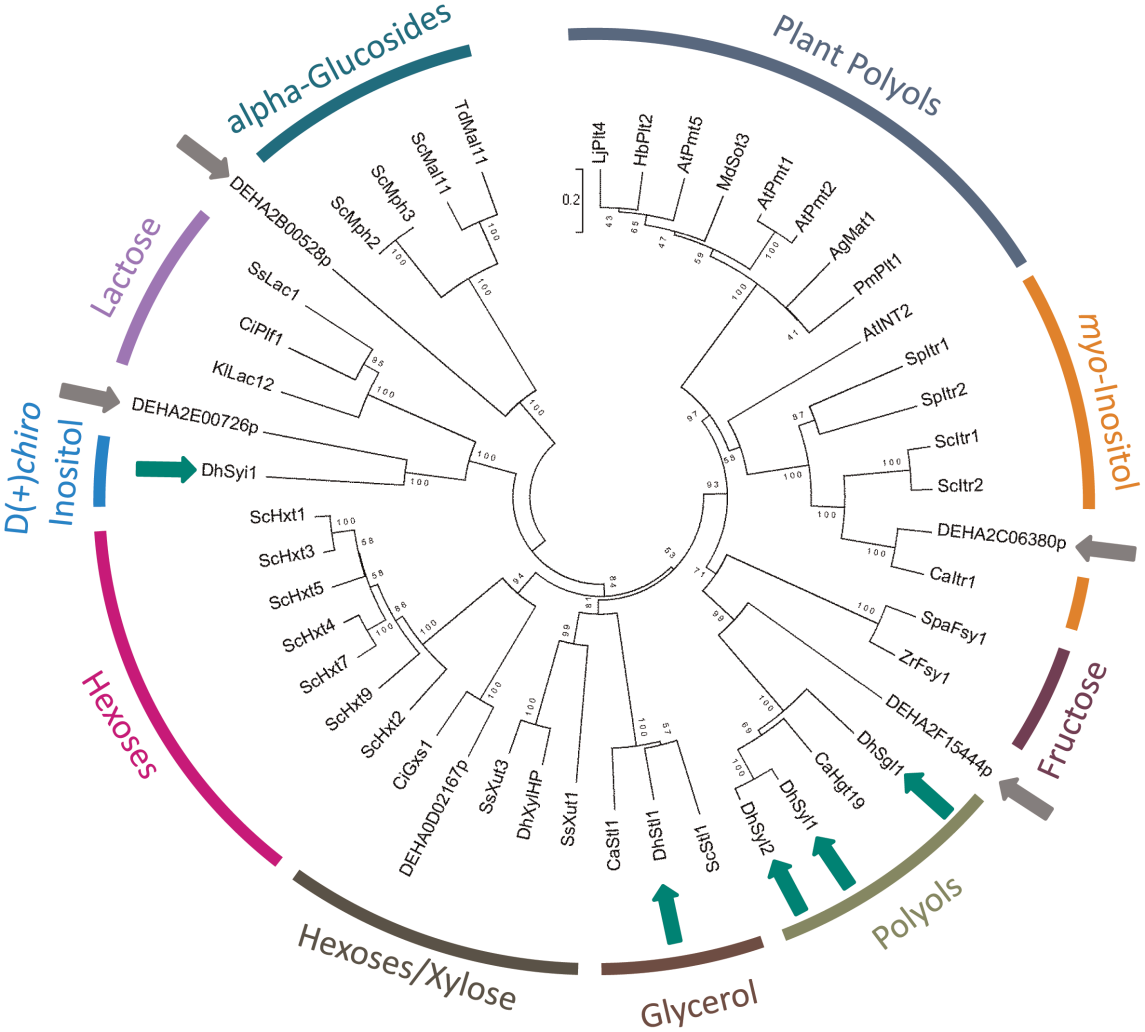
Dendrogram based on primary protein sequence homology using the neighbor-joining method (applied to 1000 bootstrap data sets). Dendrogram depicting the phylogenetic relationship between the characterized five new polyol transporters (green arrows), the four cloned putative transporters with unknown substrate (gray arrows) and other members of the Sugar Porter family. Represented proteins (and corresponding accession numbers) are: *Ag*Mat1- *Apium graveolens* mannitol transporter (AAG43998.1); *At*INT2- *Arabidopsis thaliana* inositol transporter 2 (CAJ00304.1); *At*Pmt1- *A. thaliana* polyol/monosaccharide transporter 1 (AEC06467.1); *At*Pmt2- *A. thaliana* polyol/monosaccharide transporter 2 (AEC06468.1); *At*Pmt5- *A. thaliana* polyol transporter 5 (NP_188513.1); *Ca*Itr1- *C. albicans myo*-inositol transporter 1 (XP_714885.1); *Ca*Hgt19- *C. albicans* potential *myo*-inositol transporter (XP_717819.1); *Ca*Stl1- *C. albicans* glycerol permease *Ca*O19.5753 (XP_718089.1); *Ci*Gxs1- *C. intermedia* glucose/xylose/H^+^ symporter 1 (CAI44932.1); *Ci*Plf1- *C. intermedia* putative lactose facilitator 1 (CAO79524.1); DEHA0D02167p- *D. hansenii* DEHA2D01474p hexose transporter (XP_458532.1); DEHA2B00528p- *D. hansenii* putative transporter (CAG84972.1); DEHA2C06380p- *D. hansenii* putative transporter (CAG86022.1); DEHA2E00726p- *D. hansenii* putative transporter (CAG87550.2); DEHA2F15444p- *D. hansenii* putative transporter (CAG89402.1); *Dh*Sgl1- *D. hansenii* galactitol/H^+^ symporter 1 (DEHA2E24310p; CAG88649.2); *Dh*Stl1- *D. hansenii* glycerol/H^+^ symporter 1 (DEHA2E01386p; CAG87598.2); *Dh*Syi1- *D. hansenii* D-(+)-*chiro*-inositol/H^+^ symporter 1 (DEHA2G06490p; CAG90290.2); *Dh*Syl1- *D. hansenii* sorbitol/mannitol/ribitol/arabitol/galactitol/H^+^ symporter 1 (DEHA2C05896p; CAR65543.1); *Dh*Syl2- *D. hansenii* sorbitol/mannitol/ribitol/arabitol/H^+^ symporter 2 (DEHA2C05918p; CAG86001.1); *Dh*XylHP- *D. hansenii* DEHA2C11374p hexose/xylose transporter (XP_458169.1); *Hb*Plt2- *Hevea brasiliensis* putative polyol transporter protein 2 (CAP58707.1); *Kl*Lac12- *Kluyveromyces lactis* lactose permease (XP_452193.1); *Lj*Plt4*- Lotus japonicus* putative polyol transporter protein 4 (CAJ29291.1); *Md*Sot3- *Malus domestica* sorbitol transporter (BAD42343.1); *Pm*Plt1- *Plantago major* polyol transporter (CAD58709.1); *Sc*Itr1- *S. cerevisiae myo*-inositol transporter 1 (DAA12329.1); *Sc*Itr2- *S. cerevisiae myo*-inositol transporter 2 (DAA10681.1); *Sc*Hxt1- *S. cerevisiae* hexose transporter 1 (DAA06789.1); *Sc*Hxt2- *S. cerevisiae* hexose transporter 2 (AAA34701.1); *Sc*Hxt3- *S. cerevisiae* hexose transporter 3 (DAA12185.1); *Sc*Hxt4- *S. cerevisiae* hexose transporter 4 (DAA06788.2); *Sc*Hxt5- *S. cerevisiae* hexose transporter 5 (DAA06790.1); *Sc*Hxt7- *S. cerevisiae* hexose transporter 7 (DAA12183.1); *Sc*Hxt9- *S. cerevisiae* hexose transporter 9 (NP_012316.1); *Sc*Mal11- *S. cerevisiae* maltose (alpha-glucoside) transporter (DAA08377.1); *Sc*Mph2- *S. cerevisiae* alpha-glucoside permease 2 (DAA11620.1); *Sc*Mph3- *S. cerevisiae* alpha-glucoside permease 3 (DAA08944.1); *Sc*Stl1- *S. cerevisiae* glycerol/H^+^ symporter 1 (DAA12366.1); *Sp*Itr1- *Schizosaccharomyces pombe myo*-inositol transporter 1 (CAA67211.1); *Sp*Itr2- *Schi. pombe myo*-inositol transporter 2 (NP_593320.1); *Spa*Fsy1-*S. pastorianus* fructose/H^+^ symporter (CAC08232.1); *Ss*Lac1- *Scheffersomyces stipitis* lactose permease (XP_001383110.1); *Ss*Xut1- *Sche. stipitis* sugar transporter 1 (XP_001385583.1); *Ss*Xut3- *Sche. stipitis* sugar transporter 3 (XP_001387138.1); *Td*Mal11- *Torulaspora delbrueckii* general alpha-glucoside permease (AAQ75121.1); *Zr*Fsy1- *Z. rouxii* fructose/H^+^ symporter (ZYRO0C00374p; CAR26745.1).

Phylogenetically, the plants polyol transporters form a cluster separated from the yeast transporters (Figure 6). The polyol symporters *Dh*Sgl1, *Dh*Syl1 and *Dh*Syl2 are closer to *myo*-inositol transporters *Sc*Itr1/*Sc*Itr2 and to plant polyol transporters (*e.g. Lj*Plt4) than to the glycerol symporters Stl1. The D-(+)-*chiro*-inositol symporter is phylogenetically apart from the other characterized *D. hansenii* polyol transporters.

## Discussion

While the transport of sugar compounds has been extensively studied (see [15] for a review), little information is available on transport of polyols by yeasts, although they are metabolized and/or produced by most yeast species.

Detection of polyol/sugar symport activity in *D. hansenii* CBS 767 was only possible after starving the cells. The induction of hexose/H^+^ symporters by starvation or very low substrate concentration has been frequently reported in yeasts [36], [42], [43]. In this case, it has been assumed that in general when high concentrations of glucose are available, this sugar is transported by facilitated diffusion, and that only when glucose is scarce, the H^+^ gradient is used in high-affinity symporters [19], [42]. It is conceivable that in media with high concentrations of polyols *D. hansenii* will display facilitated diffusion systems for polyols.

The presence of KCl in the symport assay made the detection of symport signal more evident. A salt-dependent behavior was already reported by Nobre *et al.* [43], for detection of sugar symport signals in *D. hansenii* INETI CL18, having been suggested that high affinity transporters could be affected by a salt-gradient (KCl or NaCl) across the plasma membrane, that influences the proton motive force, affecting the H^+^ symporter activity [43]. Also in the halotolerant yeast *Pichia sorbitophila* proton movements associated with polyols uptake was only detected in the presence of 1 M NaCl (Lages and Lucas, personal communication, 2000).

The availability of *D. hansenii* var. *hansenii* CBS 767 complete genome sequence unlocked the possibility of characterization of a broad array of new proteins. The fact that this yeast has several putative membrane transporters unrelated at the protein level with previously characterized ones, as reported by Palma *et al.* [32], prompted us to look for putative polyol transporters in its genome.

In this work we expressed nine putative membrane transporters from *D. hansenii* CBS 767 in *S. cerevisiae* YSH 1172. This yeast was previously shown to be unable to transport sorbitol [40] and we confirmed that it is also unable to transport any of the tested twelve polyols and seven sugars by a symport mechanism with protons, when grown on maltose medium.

From the nine selected putative membrane transporters, we were able to characterize the function of five of them. The galactitol/H^+^ symporter *Dh*Sgl1 (DEHA2E24310p), the sorbitol/mannitol/ribitol/arabitol/galactitol/H^+^ symporter *Dh*Syl1 (DEHA2C05896p) and the sorbitol/mannitol/ribitol/arabitol/H^+^ symporter *Dh*Syl2 (DEHA2C05918p) are located on cluster 2.A.1.1.Z23 that contains 27 members, including three *S. cerevisiae* proteins (one of them involved in vacuolar protein sorting [44]) and a putative glucose/*myo*-inositol transporter from *C. albicans* (HGT19, orf19.5447) [32]. The *myo*-inositol transporters are phlylogenetically closely related to this cluster (Figure 6). Both *Dh*Syl1 and *Dh*Syl2 have high affinity for mannitol and arabitol and low affinity for sorbitol and ribitol (Table 4).

DEHA2G06490*g* (*Dh*Syi1; strain MLY23) encodes a D-(+)-*chiro*-inositol/H^+^ symporter, with very low transport capacity. *D. hansenii* is able to transport D-(+)-*chiro*-inositol by a H^+^ symporter mechanism but not *myo*-inositol (results not shown), that is interesting since *myo*-inositol is the form most used by yeast species as growth factor, being an important cellular component in several organisms. In *A. thaliana*, the inositol transporter *At*INT2 mediates the symport of protons with several inositol epimers, including *myo*-inositol, D-(+)-*chiro*-inositol, *scyllo*-inositol and *muco*-inositol, with an intermediate affinity for *myo*-inositol [45]. In HepG2 liver cells the same protein also seems to transport both *myo*-inositol and D-(+)-*chiro*-inositol [46], contrary to the studies described so far in yeast cells, since *S. cerevisiae* transports only *myo*-inositol by proteins Itr1 and Itr2 [47] and *myo*-inositol transporters from *C. albicans* are also *myo*-inositol specific [20].

In plants, some of the already characterized polyol/H^+^ transporters also transport sugars, as the *A. thaliana At*Pmt1 and *At*Pmt2, that are designated as polyol/monosaccharide transporters and transport xylitol and fructose by a symport mechanism with protons [48]. The fructose/H^+^ symporter of *Z. rouxii Zr*Fsy1 is also able to transport xylitol [36], but the glucose/xylose/H^+^ symporter *Ci*Gxs1 of *C. intermedia* [49] did not transport any of the polyols tested in this work (MJ Leandro, unpublished results). The five *D. hansenii* polyol symporters characterized in this work do not accept sugars as substrate, although they are phylogenetically close to the *Zr*Fsy1 fructose/H^+^ symporter (Figure 6). The broad polyol spectrum *D. hansenii* carriers *Dh*Syl1 and *Dh*Syl2 also differ from plant polyol transporters as they do not transport xylitol, which is commonly transported together with sorbitol and mannitol by plant polyol transporters.

Constructed *S. cerevisiae* transformants were grown on maltose providing a positive control for detection of H^+^ symporters and avoiding repression by glucose. For transformant MLY23 we confirmed that, even using the *MET25* promoter that is irresponsive to glucose repression, D-(+)-*chiro*-inositol/H^+^ symporter activity was weaker in glucose-grown cells when compared with maltose-grown cells (results not shown), what suggests a post-transcriptional regulation by glucose.

As for the other four cloned genes, although they are being correctly expressed in *S. cerevisiae* plasma membrane, no transient alkalinization was detected for any of the putative substrates tested. The possibility that polyols are being transported by other mechanism not involving proton movements cannot be discarded.

In *D. hansenii* the CUG codon is mainly decoded as Serine but can also be decoded as Leucine (as in *S. cerevisiae*) [50], [51]. This could hamper the functionality of heterologous expression of *D. hansenii* proteins in a *S. cerevisiae* host strain, as Serine and Leucine are amino acids with very distinct properties. Analysis of the existence of CUG codons in the cloned *D. hansenii* genes (Table S1) using the software Codon Usage (Sequence Manipulation Suite, http://www.bioinformatics.org/sms2/codon_usage.html) [52], showed no relationship between the existence of CUG codons and no detection of symport activity, since DEHA2E24310*g* has two CUG codons and encodes a functional galactitol/H^+^ symporter, while DEHA2C06380*g*, DEHA2F15444*g* and DEHA2B00528*g* have no CUG codons and no symport activity was detected in the correspondent *S. cerevisiae* transformants.

To our knowledge, this is the first report on molecular characterization of yeast polyol transporters other than glycerol or *myo*-inositol in yeasts. This work contributed to the annotation of five new polyol transporters, including an unique specific D-(+)-*chiro*-inositol/H^+^ symporter, contributing to further expand the knowledge of polyol transport in yeast.

## Supporting Information

Figure S1 **Representative alignment of characterized** ***D. hansenii*** **polyol transporters.** Analysis was performed using MUSCLE web server [54] for multiple alignments. Conserved regions in the Sugar Porter family are indicated. Represented proteins (and corresponding accession numbers) are: *Dh*Syi1- D-(+)-*chiro*-inositol/H^+^ symporter (DEHA2G06490p; CAG90290.2); *Dh*Sgl1- galactitol/H^+^ symporter (DEHA2E24310p; CAG88649.2); *Dh*Syl1- sorbitol/mannitol/ribitol/arabitol/galactitol/H^+^ symporter (DEHA2C05896p; CAR65543.1); *Dh*Syl2- sorbitol/mannitol/ribitol/arabitol/H^+^ symporter (DEHA2C05918p; CAG86001.1); *Dh*Stl1- glycerol/H^+^ symporter (DEHA2E01386p; CAG87598.2).
(PDF)

Table S1 **Occurrence of CUG codons in** ***D. hansenii*** **cloned genes and predictive position of correspondent amino acid in protein topology.**
(PDF)
