# Supplementary material for: Characterization of New Polyol/H+ Symporters in Debaryomyces hansenii
Source: PLoS One. 2014 Feb 4;9(2):e88180. doi: 10.1371/journal.pone.0088180 (PMC3913770; doi:10.1371/journal.pone.0088180)
Supplement: Figure S1 — Representative alignment of characterized D. hansenii polyol transporters. Analysis was performed using MUSCLE web server [54] for multiple alignments. Conserved regions in the Sugar Porter family are indicated. Represented proteins (and corresponding accession numbers) are: DhSyi1- D-(+)-chiro-inositol/H+ symporter (DEHA2G06490p; CAG90290.2); DhSgl1- galactitol/H+ symporter (DEHA2E24310p; CAG88649.2); DhSyl1- sorbitol/mannitol/ribitol/arabitol/galactitol/H+ symporter (DEHA2C05896p; CAR65543.1); DhSyl2- sorbitol/mannitol/ribitol/arabitol/H+ symporter (DEHA2C05918p; CAG86001.1); DhStl1- glycerol/H+ symporter (DEHA2E01386p; CAG87598.2). (PDF) [file pone.0088180.s001.pdf]

Figure S1. Representative alignment of characterized *D. hansenii* polyol transporters. Analysis was performed using MUSCLE web server (Edgar, 2004) for multiple alignments. Conserved regions in the Sugar Porter family are indicated. Represented proteins (and corresponding accession numbers) are: *Dh*Syi1- D-(+)-*chiro*-inositol/H⁺ symporter (DEHA2G06490p; CAG90290.2); *Dh*Sgl1- galactitol/H⁺ symporter (DEHA2E24310p; CAG88649.2); *Dh*Syl1- sorbitol/mannitol/ribitol/arabitol/galactitol/H⁺ symporter (DEHA2C05896p; CAR65543.1); *Dh*Syl2- sorbitol/mannitol/ribitol/arabitol/H⁺ symporter (DEHA2C05918p; CAG86001.1); *Dh*Stl1- glycerol/H⁺ symporter (DEHA2E01386p; CAG87598.2).

```
               10        20        30        40        50        60        70
DhSyl1    1   MSSSAINEKPIMTDSDEANPDVGLFEPSSNSSIVEEKRRVSE-LESVHSKIAEPGQGEKGNILSQYSEKQ
DhSyl2    1   MPSETNPEKPVALRSDNPHEENGLFEHSSNSSF-NEKRRVSE-LESIHSKIAEPGQGEKGNILSQYSEKQ
DhSgl1    1   ---MKNNIDGTVSGADSIN---------------NPKEIVAESIETV-----DEHQTEEGNILAQYSEEQ
DhSyi1    1   MATEDKELGVVITDVSKLS------------------EKIEE----------QNNIISKGGSVDDYLYEK
DhStl1    1   ----------------------------------------------------MYKIWSKTNTMG--LRGK

               80        90       100       110       120       130       140
DhSyl1   70   VMQMGRNYALKYDMDPDLFASAAALARTPDEFNAMPFLSEEQKEALHKESTKKWHIPSKLIQVIALGSMA
DhSyl2   69   VMQMGRNYALKYDLDPDLFARAAALARTPDDFNAMPFITEEEKQALHKEHTKKWHIPSKLIQVIALGSMA
DhSgl1   48   TMDMGRNYGMKHGLDPELFARAAAVARSPLCFKSMKFLSEMEKSALEIEACKKWHIPRKLLEVVALGSMA
DhSyi1   43   RPWWKVNYLVRLNF------------------------------------------------CIFLITLS
DhStl1   17   PLRVAITICCTIGF-----------------------------------------------------SLF

              150       160       170       180       190       200       210
DhSyl1  140   AAVQGMDESVING--AVLFYPNYMGVGKGEYNRFGYDTDLLEGLINGAPYLCASCLAC--------WMAD
DhSyl2  139   AAVQGMDESVING--ALLFYPNYMGVGKGEYNRFGYDTDMIEGLINGAPYLCASCIAC--------WMSD
DhSgl1  118   ACVQGMDESVVNG--ANLFFPLAMGIPSMK------NYDLIEGLVNGAPYLCSS-IVC--------WTSD
DhSyi1   65   STNNGYDGSMLNGLQSVSYWPEKMGNPQGS---------VLGALSNGNVFGVVLSFVC------ASWLAD
DhStl1   34   GYDQGLMSGIITGKQFNEEFPPTHGTDQHA------------TVIQGAVTSCYE-LGCFFGALFALFQGD

                 RXGRR                         RX XX G X XXGXX XX X X PX YX X EX X XX XX
                      [2]20      230       240       250       260       270       280
DhSyl1  200   SWNRRLGRKW-TIFWTCLISAVTCF-WQGFTN-TW--YHLFIARFMLGFGIGIKSATVPAYAAECTPKQI
DhSyl2  199   LWNRHLGRKW-TIFWTCLISALTCF-WQGFTN-TW--YHLFIARFMLGFGIGIKSATVPAYAAECTPKQI
DhSgl1  171   YWNRKLGRKW-CIFWSCVITALSCI-WQGLVSLNW--FHLFIARFFMGFGMGIMTATVPAYTAETAPAHI
DhSyi1  120   KFGR-----WRCIFVGQTITILGSI-LQGVST-NY--GFFLASRIILGFGVGIAGVSSPSLISELAYPTH
DhStl1   91   KYGRRP-----MIIVGSSLIVIGTVIAVSAFGPQWGLGQFVIGRVITGLGNGMDTATIPVWQSEISKAEN

              R GX X X XX X QX X XX XG                                        P E S P RX
                      290       300       310       320       330       340       350
DhSyl1  265   RGALVMLWQFFTAFGIMLGYVACLAFYHVGDHGIGGGLNWRLMLGSACIPAIVVLFQIPFV---PESPRW
DhSyl2  264   RGALVMLWQFFTAVGIMFGYVACLAFYHVGDHGIGGGLNWRLMLGSACIPAIIVLFQIPFV---PESPRW
DhSgl1  237   RGALVMLWQLFTCVGVMLGYVSSLAFYYVGDHGISGGLNWRLTLASACIPAIFVLIQIPYV---PESPRW
DhSyi1  181   RATATTFYNVCWYLGAIIA-----AWVTYGTLNIENDYSWRIP---SYLQAALPCLQIAFFWMVPESPRY
DhStl1  156   RGLLVNLEGSMVAVGTFIAY-----WLDFGLSYVDTSVQWRFPVAFQIVFALFLFLGVAQL---PESPRW

              L
                        360       370       380       390       400       410       420
DhSyl1  332   LMGKDRHKDSYDSLCQLRL-DKIAAARDTFYQYVLLQEEASYQ-----GIPTWKR----LVEMFTIRRNR
DhSyl2  331   LMGKDRHKDSYDSLCQLRL-DKIAAARDTFYQYVLLQEEASYQ-----GIPTWKR----LVEMFTIRRNR
DhSgl1  304   LMGKHRHRDAFDSLKILRH-SEIEAARDCFYQYVLLSEEGSYV-----GVSFYKR----VIEMFSIRRNR
DhSyi1  243   LINKDRISDAEKVLRKFHAGGETHERANDLVSFEIEEIQAALEIEKLSSNSRYSD----FITMPTFRK--
DhStl1  218   LIAHGLKDEAHYVLATLND-VDIDD------EFVIEESAIITD-----GVNRFARTQIGFKELFSGGKQQ

                      [LI] QX X QQX [ST][GN] X XX Y Y F
                        4[illegible]                          460       470       480       490
DhSyl1  391   ---NGALGAWIVMFMQQFCGINAIVYYNSTIF-IRGGFTEVAALTASWGFGMVNFLFAIPTFYTI-DTFG
DhSyl2  390   ---NGALGAWIVMFMQQFCGINAIVYYNSTIF-IQGGFTEVDALTASWGFGMVNFLFAIPTFYTI-DTFG
DhSgl1  363   ---NAALGAWIVMFMQQFCGINVISYYSSSIF-VESDLSEIKAMLASWGFGMVEVISCIPTFYTI-DSVG
DhSyi1  306   ---RLFLAVFVAVIM-QLSGNGLVSYYLNKVL-NSIGITDPHEQLKINGCLMVYNLVISSVVASVAGRFK
DhStl1  276   NFARMIIGA-STQFFQQFTGCNASIYYSTVLFENSIGLTGKLPLILGGVFATIYALSTIPSFFLI-DRLG

                        500       510       520       530       540       550       560
DhSyl1  457   RRNLLLLTFPLMSIFL-LWTGFGFLASND---QTRIG-IVASGVYIFSAIYSAGEGPVPFTYSAEAFPLY
DhSyl2  456   RRNLLLFTFPLMALFL-IWTGLGFVAEDGT--KTQTG-IVASGVYIFSAIYSAGEGPVPFTYSAEAFPLY
DhSgl1  429   RRNLLLATFPLMSLSL-LMTGFGFWIPEGTSRDGRLA-CITIGIYSFAIFYNFGEGPIPFTYSAEAFPLY
DhSyi1  372   RRSMFLICTILMLVFYVIWTILSAINQQRNFEQKSLGNGVIAMIFFYYLAYDIGANGLPFLYITEIMPYS
DhStl1  344   RRALFLIGATGQGISF-TIT-FACLIPDNGQNKETAK-GAAVGIFLFIVFFAFTILPLPWIYPPEINPLR

                                                                     P E T KG X XX E
                        570       580       590       600       610                 630
DhSyl1  522   IRDLGMGFATATCWFFNFILALTWPKLVSSFTSTGAFGWYAAWNIVGFFLVLWFLPETKGLTLEELDEVF
DhSyl2  522   IRDIGMGFATATCWFFNFILALTWPKLVSSFGRTGAFMWYAAWNIAGFFMVLWFLPETKGLTLEELDEVF
DhSgl1  497   IRDLGMGFATSTCWFFNFILAFTWPRLKNAFRSQGAFGWYAAWNIVGFFLVLWFLPETKGLTLEELDDVF
DhSyi1  442   HRAKGMNIFTVSQNIIIIFNGFVNPIAMDAI-EWKYYIVYCCILAVEVITVLFTFVETSAYTLEEVAKVF
DhStl1  411   TRTVATAVSTCTNWLTNFAVVMFTPIFIGAS-SYGCYLFFAIMNFLFIPVIFWFYPETAGRELEEIDIIF

                        640       650       660       670       680       690       700
DhSyl1  592   SVS-TRKHAKWQTKHFLNGIQ---------RVVLRRHIDPLPPLYEHQKRLAVTNPDWNNKTETAHIE--
DhSyl2  592   SVS-TRKHAIYQTKSILNSFQ---------RVVLRRHIDPLPPLYEHQKRLAVTNPDWNNKTETAHIE--
DhSgl1  567   SVS-LRKHAHFRTKEFVRGLN---------KYVLKKEVEPLNPLYMHT-RLAVTNQKWNDKTTILHEEGI
DhSyi1  511   GDD--------------PGQT-------------LIHLSSAPP-----------------KPEVGHIETV
DhStl1  480   AKAYVDNRLPWRVAATLPHLNFKEQEQEGIKLGLYDDFEKEAPEHVNTLSDASGSEDGSNEAASVKPAEV

DhSyl1  649   -
DhSyl2  649   -
DhSgl1  626   S
DhSyi1  536   -
DhStl1  549   -
```

Supplementary references

Edgar, R C (2004) MUSCLE: multiple sequence alignment with high accuracy and high throughput. *Nucleic Acids Res* 32: 1792–1797.
