# Supplementary material for: Characterization of New Polyol/H+ Symporters in Debaryomyces hansenii
Source: PLoS One. 2014 Feb 4;9(2):e88180. doi: 10.1371/journal.pone.0088180 (PMC3913770; doi:10.1371/journal.pone.0088180)
Supplement: Table S1 — Occurrence of CUG codons in D. hansenii cloned genes and predictive position of correspondent amino acid in protein topology. (PDF) [file pone.0088180.s002.pdf]

Table S1. Occurrence of CUG codons in *D. hansenii* cloned genes and predictive position of correspondent amino acid in protein topology.

***D. hansenii* genes**	**Number of CUG codons**	**Position of correspondent amino acid**	**Correspondent *S. cerevisiae* strain**	**Polyol/H^+ symport activity detected (protein name)**
DEHA2C06380*g*	**0**	**-**	MLY7	-
DEHA2E01386*g*	**1**	TMD 3	MLY8	Glycerol (*Dh*Stl1)
DEHA2C05896*g*	**1**	N-terminal	MLY9	Sorbitol/mannitol/ribitol/arabitol/galactitol (*Dh*Syl1)
DEHA2C05918*g*	**0**	**-**	MLY12	Sorbitol/mannitol/ribitol/arabitol (*Dh*Syl2)
DEHA2F15444*g*	**0**	**-**	MLY20	-
DEHA2E24310*g*	**2**	N-terminal; TMD 8	MLY22	Galactitol (*Dh*Sgl1)
DEHA2G06490*g*	**1**	Outside, between TMD 1 and TMD 2	MLY23	D-(+)-*chiro*-Inositol (*Dh*Syi1)
DEHA2E00726*g*	**1**	TMD 10	MLY25	-
DEHA2B00528*g*	**0**	**-**	MLY26	-

(TMD – transmembrane domain)
